# Origin of Polar Order in Dense Suspensions of Phototactic Micro-Swimmers

**DOI:** 10.1371/journal.pone.0038895

**Published:** 2012-06-19

**Authors:** Silvano Furlan, Diego Comparini, Marzena Ciszak, Lucia Beccai, Stefano Mancuso, Barbara Mazzolai

**Affiliations:** 1 Center for Micro-BioRobotics, Istituto Italiano di Tecnologia, Pontedera, Italy; 2 The BioRobotics Institute, Scuola Superiore Sant’Anna, Pontedera, Italy; 3 Laboratorio Internazionale di Neurobiologia Vegetale - Department of Plant Soil & Environmental Science, University of Florence, Florence, Italy; 4 Consiglio Nazionale delle Ricerche - Istituto Nazionale di Ottica, Florence, Italy; Tel Aviv University, Israel

## Abstract

A main question for the study of collective motion in living organisms is the origin of orientational polar order, i.e., how organisms align and what are the benefits of such collective behaviour. In the case of micro-organisms swimming at a low Reynolds number, steric repulsion and long-range hydrodynamic interactions are not sufficient to explain a homogeneous polar order state in which the direction of motion is aligned. An external symmetry-breaking guiding field such as a mechanism of taxis appears necessary to understand this phonemonon. We have investigated the onset of polar order in the velocity field induced by phototaxis in a suspension of a motile micro-organism, the algae *Chlamydomonas reinhardtii*, for density values above the limit provided by the hydrodynamic approximation of a force dipole model. We show that polar order originates from a combination of both the external guiding field intensity and the population density. In particular, we show evidence for a linear dependence of a phototactic guiding field on cell density to determine the polar order for dense suspensions and demonstrate the existence of a density threshold for the origin of polar order. This threshold represents the density value below which cells undergoing phototaxis are not able to maintain a homogeneous polar order state and marks the transition to ordered collective motion. Such a transition is driven by a noise dominated phototactic reorientation where the noise is modelled as a normal distribution with a variance that is inversely proportional to the guiding field strength. Finally, we discuss the role of density in dense suspensions of phototactic micro-swimmers.

## Introduction

Independent of length scales, collective motion phenomena of many biological systems result in a polar order of the direction of motion in which the velocities of moving entities are directionally aligned. Animal herds align to the same orientation and escape in a coherent direction in the presence of a predator in a way that is similar to human crowd dynamics in panic escape [1], [2]. Fish schools, bird flocks and insect swarms exhibit polar alignment during their motion as a response to external influences [3], [4]. At the microscale, cellular motion and growth in polar ordered tissues such as epithelia are fundamental to their function of resisting mechanical stresses [5]. Similarly, coherent directional movement of micro-organism blooms attempts to achieve optimal light or nutrient uptake to satisfy the energy needs of the organisms or increase their reproductive success [6]. In each of these examples, a coherent direction of motion arises in aggregations and clusters of living species, underlining a strict relationship between population density and the ability of the population to respond coherently at a defined strength of an external stimulus. This suggests that the aggregation of a specific cluster density plays a key role in determining the presence of a polar order response, which may result in an evolutionary advantage [3].

For both biological micro-organisms and artificial active particles [7] swimming at a highly viscous limit (at a low Reynolds number), the interactions involved in the swimming motion are affected by density, which can alter both the ability of individuals to track the guiding field as well as their hydrodynamic flow field. The tracking ability consists of a reorientation mechanism, called taxis, that is able to turn the swimming particle towards an externally determined direction. Taxis is a necessary condition for a homogeneous polarised state of the velocity flow field [8], and steric repulsion and long-range hydrodynamic interactions alone are insufficient [9], [10]. For the unicellular phototactic organisms such as the archetypal green alga *Chlamydomonas reinhardtii*, reorientation originates from the cell’s ability to track a light gradient in both the positive and negative direction according to light intensity. This mechanism is a direct consequence of the helical trajectory followed by the organism [11], resulting from a non-perfectly planar motion of the cell’s flagellar stroke. This behavior is critical to phototaxis [12]. However, when the organism is present in dense suspensions, the helical path that is necessary for the organism to track the light faces some motion constrains due to a reduced distance among cells. Additionally, the hydrodynamic interactions that describe the effects of the swimming object on the surrounding flow field change at shorter distances. In diluite suspensions, the hydrodynamic flow field induced by *C. reinhardtii* has been attributed to a stresslet flow [13]. This is a fundamental solution of the Stokes equations that is able to describe the flow created by the swimmer and the perturbations affecting the nearby cells. This solution is represented by an effective force dipole model, which indicates that the hydrodynamic effects are responsible for the orientational order of the velocity field [13], a characteristic commonly shared by the so called pullers swimming particles. When population density increases the approximation to a force dipole model loses validity [14], leaving the question of the polar order in this density condition open.

Beyond the complexity of the micro-organism motion, the two approaches of self-propelled particle models [15] and continuum theory [9], [10], [16] were used to provide insights into the mechanisms responsible for the spontaneous emergence of collectively oriented motions in terms of the swimmer activity. A theoretical model describing the onset of polar order and its dependence on the population density in the absence and the presence of an external field was derived in Ref. [17]. Here, we address this problem through the experimental investigation of the onset of polar order by considering a population of micro-organisms. In particular, we consider the unicellular biflagellate green alga *C. reinhardtii* as a representative case study.

**Figure 1:**
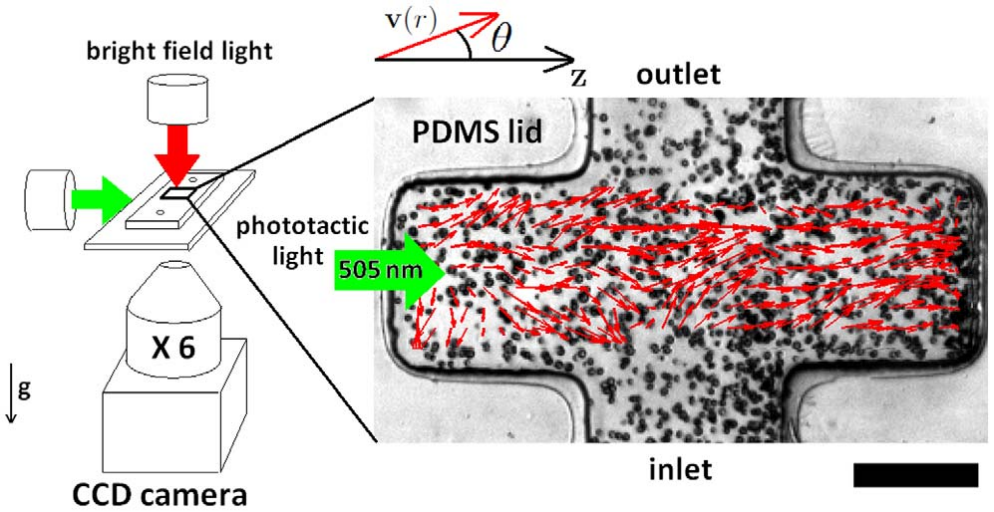
Experimental system. Photo-movement assay conducted using bright field microscopy (left). A lateral green light source is used to obtain a photophobic response from an algae population swimming in the PDMS microfluidic channel. (right) PIV velocity field (red arrows) extracted from two consecutive frames, superimposed on the correspondening movie snapshot showing the spatial distribution of the algae (scale bar  = 200 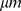). The polar angle 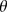 represents the orientation of the velocity field vectors 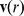 with respect to the phototaxis gradient direction 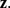

This study uses a photo-movement assay (see Fig. 1) to study the effect of an external phototaxis stimulus at density ranges above the limit of the force dipole model, which was previously proven to vanish for a distance less than 7 times algae radius [14]. The velocity field data extracted through a cross-correlation particle image velocimetry (PIV) showed that in the presence of phototaxis, a homogeneous polarised state develops with a linear increase in cell density. Based on an existing minimal model elaborated for noisy driven reorientation [16], we obtained a density threshold under which polar order is not sustained. Finally, we discuss some hypotheses of the role of density in the phototactic mechanism and the possibility of a general rule linking the onset of polar order to the population density of clusters formed by living organisms.

## Results

### Characterisation of Micro-swimmers Motion in Microfluidic Channels

**Figure 2:**
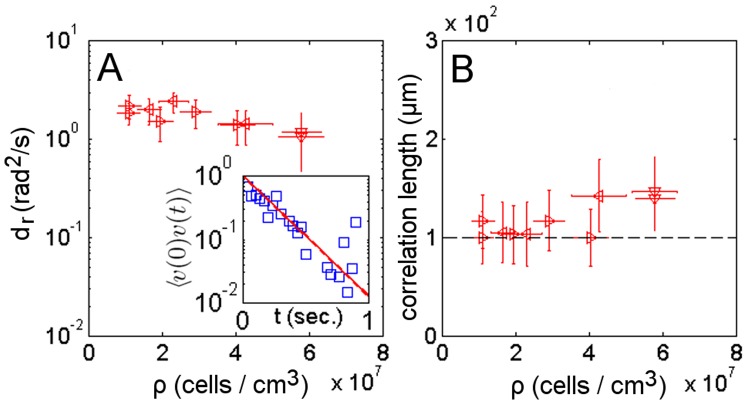
Temporal and spatial decorrelations. The orientational coefficient 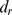 (A) and correlation length (B) as a function of cell suspension density for samples run in the dark. Samples from the same culture share the same mark. (inset) Time autocorrelation function of the velocity direction used to extract 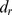 value.

To validate that the microfluidic setup used for the photo-movement assay experiments does not influence the swimming motion of the organisms, we characterise the motion of the micro-swimmers in terms of the rotational and spatial diffusion coefficients. Both these coefficients capture the reorientation in time and space of the PIV extracted velocity field **v**. The rotational diffusion coefficient 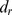 is determined from the time autocorrelation function of the velocity direction 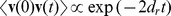 (see Eq. 5 in Methods). For dense suspensions in the absence of phototaxis, 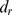 shows a plateau (Fig. 2A) with values that are comparable with previous measurements (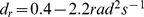) [18]. The spatial diffusion of the swimmers is measured by the correlation length, estimated as the first zero crossing of the spatial correlation function 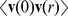 (see Eq. 6 in Methods and Fig. 2B). Given that the correlation length is similar to the dimensions of the microfluidic channel, the boundary effects on the swimmers’ velocity cannot be completely neglected. The velocity of the swimmers decreases with respect to their absolute velocity measured in free media because the viscous drag increases as the swimmer body comes closer to the boundary [13]. The scaling of velocity with viscosity is usually described with a boundary correction factor [19], which seems to be constant with density as shown by the plateau of the correlation length (Fig. 2B). The spatial correlation is also related to the decay length of polarisation fluctuation, which for rod-like swimming particles of length 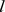 is estimated to be 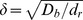, where 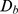 is the bend diffusion constant 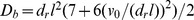 and 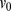 is the self-propulsion velocity [9]. For the sake of simplicity, the space occupied by the *C. reinhardtii* body and the envelope of its flagellar stroke can be approximated as a rod-like shape, for which the expected length of polarisation fluctuation is 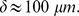. This estimation is consistent with the extracted experimental correlation length (Fig. 2B dashed line).

**Figure 3:**
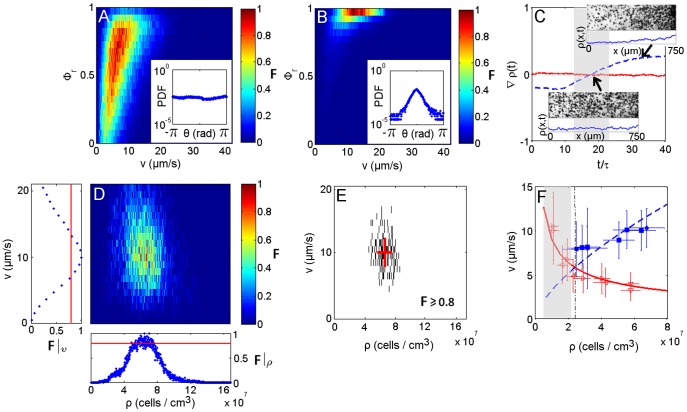
Dynamic characteristics of phototactic micro-swimmers. A correlation diagram between the coherent direction motion parameter 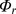 and the modulus of the velocity field both in the dark (A) and light (B); respective distribution histograms of the polar angle 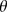 representing the velocity field orientation (insets). The colour bar indicates the normalised frequency of event occurrences F. (C) Cell density gradient 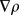 along the main channel axis 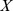 versus time of samples run in dark (solid line) and light conditions (dashed line), scaled to the orientation fluctuation 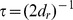 to give the number of statistically independent configurations; images and density distributions related to a homogeneous cell suspension (lower inset) and cell accumulation (upper inset). (D) Correlation diagram collecting event occurrences for a given combination of cell density and velocity for a single sample. Correlation diagrams were obtained by coupling the velocity and density field elements according to the interrogation window and counting the event occurrences for each pair of elements over all movie frames. The normalised value of occurrences of the couples defines the local frequency of event occurrences F. Lateral subplots represent the cumulative occurrences for variable density 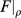 and velocity 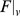. The solid line corresponds to occurrences 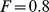. (E) Scatter plot obtained by a threshold filter of the correlation frequencies of the velocity - density plot, with 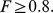 Data were fit with a Gaussian distribution. Extracted means and standard deviations are reported on the scatter plot (red solid line). Velocity as a function of density (F): collected data are fit with kinetic curves in the dark (hollow marks and solid line) and light (filled marks and dashed line); the intersection of both kinetic curves marks the density threshold for the onset of polar order 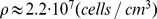 (dashed-dot line).

Phototaxis studies clearly show that the presence of light results in a steering response causing reorientation of the cell with respect to the direction of light [18]. This phenomenon was employed in the movement assay to obtain action spectroscopy of photo-movement in a phototaxis population method [20], where the negative phototaxis was used to obtain the swimming rate by measuring the movement of a shock wave inside the suspension. The flow perturbation by swimmers on their neighbours during phototaxis has not yet been investigated. For this purpose, we measure the level of coherent directional motion in the velocity field using the order parameter 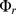 (see Eq. 8 in Methods). The collection of the local frequency of event occurrences F in a correlation diagram for a given combination of speed and 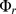 evaluated on a radius 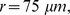 an order of magnitude wider than the cells, exhibit two different scenarios. In the dark the order parameter is in the range 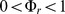 and indicates an absence of organisation of the velocity vectors in neighbouring regions (Fig. 3A). This results in a homogeneous spatial and temporal distribution of cell density (Fig. 3C lower inset and solid line). On the other hand, phototaxis induced motion results in 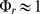 (Fig. 3B), suggesting nearly parallel velocity vectors inside the region r with a highly oriented probability distribution function (PDF) of the polar angle (Fig. 3B inset). This orientation coherence results in a net-mass transport during phototaxis, causing density and velocity fluctuations and accumulation at one end of the channel (Fig. 3C upper inset and dashed line, Video S1). To avoid these boundary phenomena, data analyses of phototactic samples were performed in a time frame in which the spatial cell density gradient 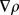 approaches zero (Fig. 3C gray region).

### Dependence of Velocity on Density

To study swimmers dynamics, the local frequency of event occurrences F for a given combination of cell density and velocity was collected in the correlation diagram shown in Fig. 3D. For each sample, a scatter plot in Fig. 3E was obtained by considering significant events that satisfy 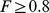. The scatter plot was fitted with a Gaussian distribution. The mean and deviation values were reported in the velocity-density graph (Fig. 3F). Two different patterns of kinetics were observed between dark (solid line) and light (dashed line) conditions. This difference arises above a density value where the approximation of the swimmer by a force dipole model is no longer valid. To estimate the limit density, we use the results reported in [21] regarding the mean distance of a random distribution of cells. Morover, we consider the upper limit marked by the effect of hydrodynamic interactions modelled in terms of the volume exclusion of a sphere of radius R, representing the interaction distance. Then, the limit density is given by:
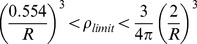
(1)which, for a validity limit of the force dipole model defined as 7 times the algae radius [14], is 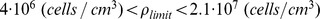 (gray region in Fig. 3F). In the dark, swimmer velocity is inversely proportional to cell density. This phenomenon can be understood by considering that the spatially random distribution of cells and their uniform polar angle distributions (Fig. 3A inset) are driven by dispersion effects that are well described by a Poisson statistics. The mean distance 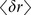 between nearest neighbour particles in a planar projection of randomly distributed particles has been estimated as 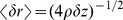 [21], where 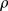 is the cell density and 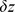 the depth of field. The corresponding particle velocity is obtained by rescaling the mean distance to the sampling frame rate. For our system parameters, the theoretical curve given in [21] fits the data inform in dark condition (Fig. 3F solid line). Another model in the literature describes the phenomenon of a reduction of the mean-free path and velocity with an increase in density [22]. In both cases discussed in Ref. [21], [22], the dynamics of C. reinhardtii in dark condition for density values above the validity limit of the force dipole model defined in Eq. (1) seems to retain pullers swimmer type characteristics [23]. With phototaxis the proportionality is reversed, with velocity increasing with density. The resulting collective velocity was modelled by applying flux conservation of directionally aligned swimming organisms [24] and yielding the relation 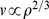 to fit the data (Fig. 3F dashed line).

### Dependence of Polar Order on Density

**Figure 4:**
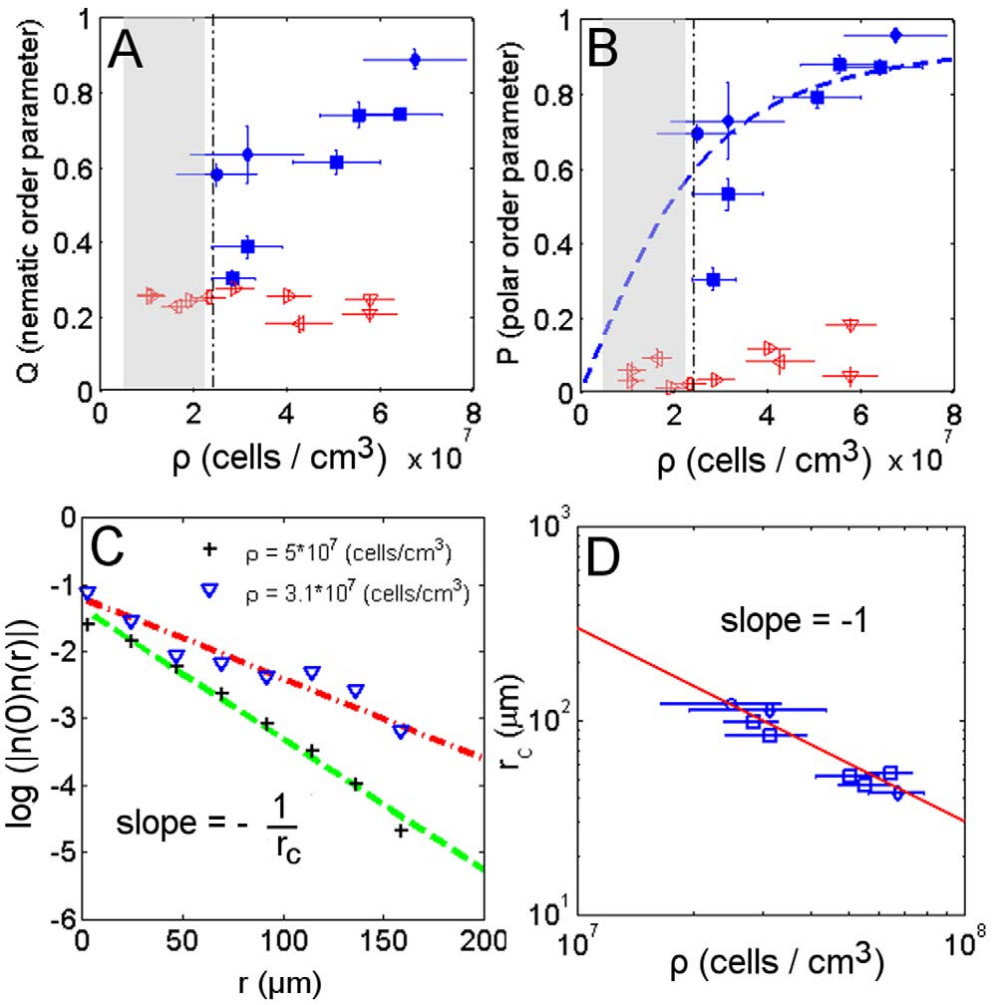
Order parameters and fluctuation dependence on density. Nematic order parameter (A) and polar order parameter (B) as a function of cell density in dark (hollow marks) and phototaxis conditions (filled marks). Linear dependence of the guiding field strength on the suspension density 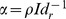 fit with the theoretical estimation of the average cosine (dashed line) with 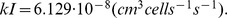 For phototactic experiments near the density threshold (dashed-dot line), both orientational order parameters are reduced. (C) Spatial correlation of the fluctuation coordinate 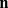 for two different density samples. The higher density condition features a higher slope. (D) The inverse of the slope of the spatial correlation of the fluctuation, corresponding to the correlation radius of fluctuation 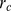 is plotted against each sample suspension density. The slope of the logarithmic plot shows the exponent order of the relationship between the correlation radius of fluctuation 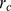 and density 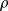 to be 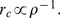

The increasing collective velocity and the highly coherent directional motion observed in dense suspensions of phototaxis experiments arise from phototaxis induced reorientation. The dependence of this phenomenon on suspension density is investigated here through an orientational order parameter for the velocity field. As for liquid-crystalline materials [25], the evolution of the orientation field may in our case be quantified by the nematic order parameter 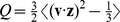 and the polar order parameter 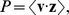 where 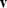 is the swimmer velocity field director and 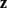 the nematic director denoting the direction of broken symmetry. Together these terms describe the possibility of isotropic (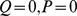), nematic (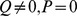) or polar state (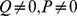) of swimmers’ velocity field. The presence of an external guiding field, in our case represented by phototaxis, fixes the nematic director 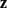 to the maximum light gradient. In the absence of light, the system is in an isotropic state with nearly null nematic and polar order parameter values (Fig. 4 hollow marks). On the other hand, a polar state is observed during phototaxis due to increases in both the polar and nematic order parameters (Fig. 4 filled marks). We access the dependence of the orientational order on density by considering the polar order parameter in terms of the average cosine of the polar angle 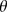. Gruler et al. [26] obtained an estimation of the average cosine in terms of the guiding field strength 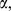 describing the distribution of cell orientation in the presence of an aligning guiding field, 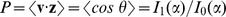, where 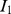 and 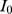 are modified (hyperbolic) Bessel functions of first and zero order, respectively. The guiding field strength 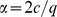 is defined as the ratio of the deterministic torque and the stochastic torque influencing swimmers orientation. The stochastic noise intensity 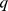 is related to the rotational diffusion 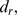 which remains constant over the analysed density range as demonstrated previously. Under phototactic conditions, the guiding field strength is reported to be proportional to the intensity of light 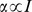 [27]. For a fixed phototactic light intensity, the guiding field strength also depends on the suspension density 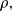 resulting in an increasing orientational order with density, as observed in Fig. 4. The best fit of the experimental polar order parameter with the estimation of the average cosine results in a guiding field that is well approximated by the first order polynomial relation 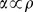 (Fig. 4B dashed line).

Two additional approaches were used to show the linear relationship between the guiding field strength and density, namely considering the spatial correlation of the velocity field fluctuation and the angular distribution of the velocity field. In the former approach the fluctuation coordinate 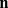 is defined to be orthogonal to the nematic director 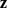 and is represented by the difference between the swimmers velocity director 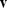 and the nematic director 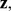 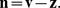 In the presence of a guiding field, the spatial correlation of fluctuations 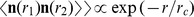 was shown to decrease exponentially with the distance 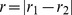, with the correlation radius defined by the constant rate 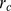 (Fig. 4C). In particular, the correlation radius is related to the guiding field strength through 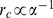 [28]. The assumption of linearity between the density and the guiding field implies that the correlation radius decreases with the same exponent order in both cases. This is confirmed in Fig. 4D where 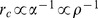.

**Figure 5:**
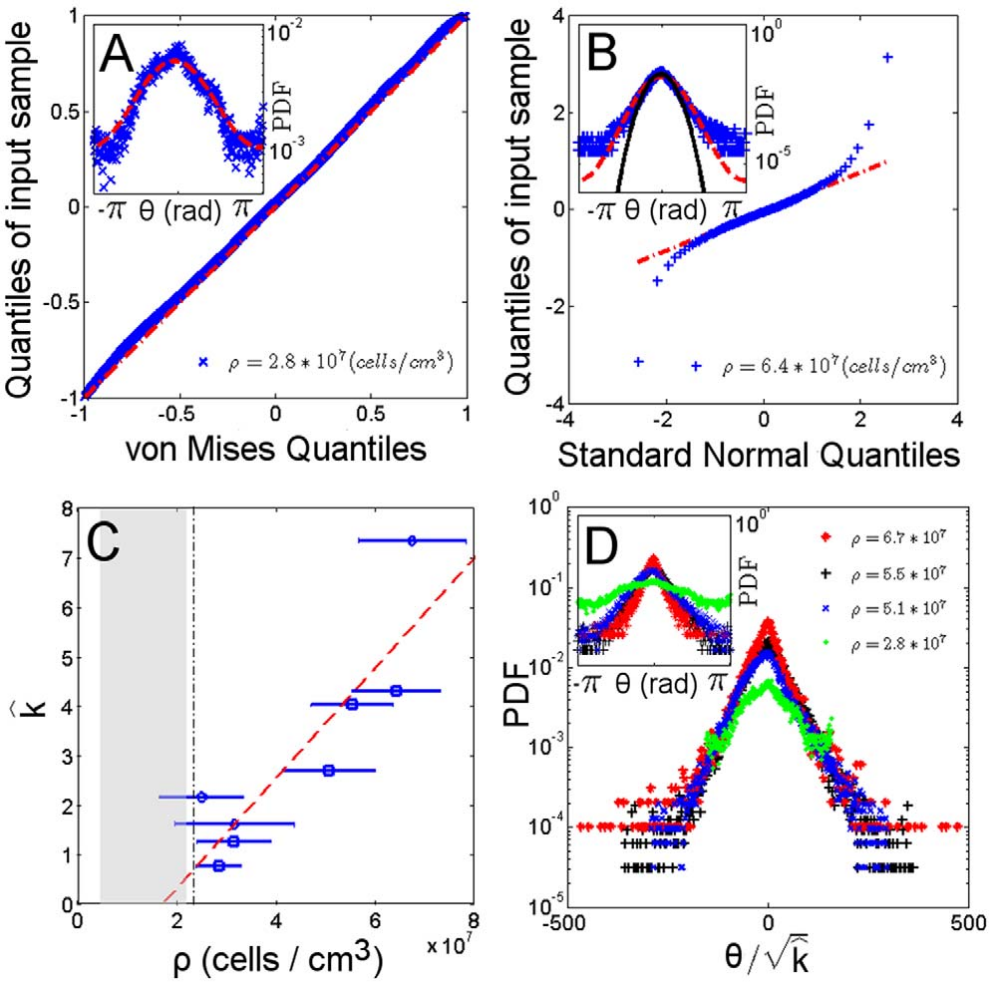
Orientation distribution. (A) Q-Q linearised plot of the orientation field using a goodness of fit method. Data from a von Mises distribution are plotted along the dashed-dot line. (inset) A von Mises probability density function (PDF) (dashed line) fit of the orientational data distribution. (B) Q-Q plot of the sample quantiles versus theoretical quantiles from a normal distribution. Dense suspension samples exhibit a linear relationship (dashed-dot line) in a region corresponding to the small angles near the polar direction 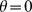 (inset). When the orientation distribution lies in an arc of sufficiently small length near the polar direction, the sample data (cross point), von Mises (dashed line) and standard normal (solid line) PDF are well approximated. The finiteness of the data is responsible for the tail departure at higher angles. (C) The estimated concentration parameter 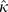 versus the density of the cells suspension 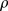 shows a linear relationship (dashed line). The condition 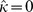 occurs for a suspension density next to the density threshold (dashed-dot line). (D) The orientational distribution of samples with different density (inset) shows a data collapse when rescaled to the square root of the estimated concentration parameter 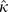. In fact, for sufficiently high 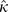, orientational data are approximated to a normal distribution, with the standard deviation 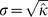 as a scaling factor.

The second approach to demonstrating the linear relationship uses the circular distribution of von Mises 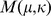 (alternatively called normal circular distribution) to describe angular distribution [29]. Here, the parameter 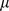 is the mean direction of the population while 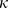 is known as the concentration parameter, describing the concentration of data towards the population mean direction 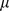. The Q-Q linearised plot of the angular distribution of the sample orientation shows that the experimental data follow the dashed line in Fig. 5A indicating the consistency of the driving stochastic process with a von Mises distribution. Using the same notation as in Ref. [29], for a suspension of n cells the velocity field can be described with a complex mean field description through 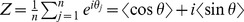. This expression decomposes the velocity direction 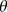 into the real and imaginary parts of the complex mean field Z, corresponding to the nematic coordinate 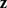 and the fluctuation coordinate 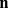, respectively. The first trigonometric moment of a von Mises distribution has a null imaginary part 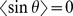 accounting for the absence of velocity field fluctuations and a real part 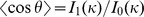 that corresponds to the previously defined polar order parameter P. Note that the guiding field strength 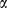 assumes the form of the concentration parameter 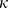 of the distribution. An estimation of the concentration parameter 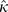 was obtained through a maximum likelihood method from each data sample. Plotting this parameter against density again demonstrates a linear relationship with the guiding field strength (Fig. 5C). The condition of a null concentration parameter 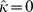 is obtained at a density value of 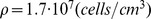. The 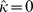 condition matches the density limit of a force dipole description of the swimmer expressed in Eq. (1) (Fig. 5C grayed region). In the case of sufficiently large 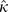 value, the von Mises distribution can be approximated by a normal distribution 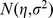 of mean 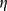 and standard deviation 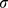, where 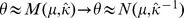 for 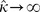 [30]. This approximation is valid in the region of small angles between the velocity vector 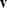 and the nematic director 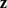 and for dense suspensions (Fig. 5B). As a result of the approximation to a normal distribution, the rescaling of the angles in terms of the square root of the estimated concentration parameter leads to a data collapse of the angular probability distribution, as shown in Figure 5D. Using normal distribution results for suitable large 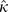 values, the imaginary part of the velocity mean field representing fluctuations 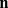 can be obtained as
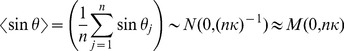
(2)so that the mean field fluctuations can be modelled as a null average normal noise with a variance that depends on density. In particular, the concentration parameter of the von Mises process describing the data is scaled by a density dependent term n accounting for the number of cells, where 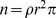 and 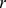 is the interaction radius. Equivalently, the guiding field strength has the form 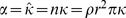. Considering the radius of interaction as a fixed property of cells, the number of the nearest neighbours affects cells’ ability to reorient by decreasing fluctuation variance. In a similar way, the Vicsek model updating rule [15] that drives the reorientation of the velocity directions of self-propelled particles is affected by the local density of particles within a circle of interaction radius r, and the resulting onset of order was demonstrated to appear over a critical density.

### Density Threshold for Order Onset

For the high density regime above the force dipole model approximation, it can be assumed that the phototactically driven mechanism aligning a single cell is affected by noisy interactions with neighbouring swimmers. Bertin et al. [16] considered a specific noisy alignment rule for pairwise interactions of self-propelled point particles and found a density threshold for the emergence of an aligned motion, defined as
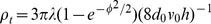
(3)where 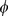 is a noise dependent reorientation parameter, 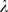 is the reorientation probability per unit time, 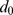 the interaction range, 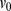 the particle velocity and 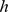 the suspension thickness. Based on the above noisy aligning model, we introduce an external field term that influences the noisy driven alignment term 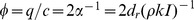, where k is a geometrical factor taking into account the area of the cell that is sensitive to exposed light and the investigated volume. This noisy term accounts for the stochastic nature of the *C. reinhardtii* motion that originates from the complex hydrodynamic phenomena [14], internal biochemical noise [31] and other sources of noise influencing phototaxis [12]. In the case of strong phototactic conditions, reorientation rate 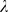 can be approximated by the deterministic part 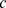 of the guiding field strength 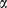 as shown in Ref. [26]. When truncating the exponential term in Eq. (3) to a first order Taylor expansion, the density threshold becomes
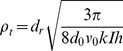
(4)where 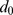 is equal to the decay length of polarisation fluctuation 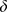. Introducing the values corresponding to the phototaxis condition (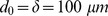, 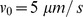, 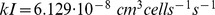, 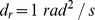, 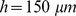), the density threshold 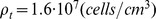 is obtained. The estimated magnitude of the threshold is of the order of the density corresponding to the intersection point between the velocity-density curves in dark and light conditions (Fig. 3D dashed-dot line). Furthermore, the estimated threshold is consistent with the density value of a null concentration parameter obtained from Fig. 5C. The threshold represents the density below which cells exposed to negative phototaxis are not able to maintain a homogeneous polar order state, and marks the transition to an ordered collective motion with this density exhibiting a linear dependence on the guiding field strength. The similarity of the estimated threshold to the analysed data confirms the assumption of a noise dominated phototactic reorientation that could be explained by considering the presence of a hydrodynamic horizon over which simple hydrodynamic effects are no longer sufficient to explain the ordering phenomena and thus noise interactions should be included [32].

## Discussion

Experimental studies of micro-swimmers in dense suspensions have shown that besides the light intensity, another variable accounts for the onset of polar order, namely, the density of cells. We have demonstrated that the polar order of the velocity field depends linearly on the density. This dependence is due to nearest neighbour interactions that dominate in dense suspensions. The experiment conducted in the dark demonstrated an isotropic velocity field, even though simulations in the absence of an external field have suggested a tendency to develop an anisotropic state due to hydrodynamic interactions [33]. To explain this discrepancy, we considered the addition of noisy fluctuations that prevent the swimmers from developing polar order in dark conditions. Conversely, in the presence of a phototactic field, the noisy fluctuations in the velocity field are reduced, leading to coherent directional motion. To analyse this situation we considered an existing minimal model elaborated for a noisy driven reorientation mechanism where the phototactic guiding field tunes the noisy driven alignment term. From this model we derived a density threshold below which cells exposed to negative phototaxis are not able to maintain homogeneous polar ordered motion due to the increasing distance 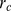 of the spatial correlation of fluctuations 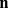 at low swimmer density (Fig. 4C). The theoretically estimated magnitude of the density threshold is of the same order as that corresponding to the intersection point between the velocity-density curves and to the condition of an experimentally obtained null concentration parameter 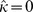. For higher densities, the reduced distance between the nearest cells causes interactions to play a dominant role in the origin of polar order, as suggested in Ref. [13]. We have shown that the effect of these interactions on the velocity field direction in the case of a strong phototactic condition can be described by a normal distribution with a variance that is inversely proportional to the guiding field strength.

The density dependence of the guiding field strength for values above a density threshold drives the swimmers towards the onset of polar ordered motion, but this issue of whether the source of this ordering is only mechanical or also chemical requires further investigation. Mechanical terms for dense suspensions include the complexity of the multipole expansion in the hydrodynamics of the swimmers [14] and their steric effects, with the latter being insufficient to yield a homogeneous polar state [9], [10]. The mechanical interaction at reduced distance may lead to synchronisation between oscillating biological systems that are hydrodynamically coupled, such as flagella [34], [35] and cells [36], [37]. Experiments [31], [35], [38] have explored the phenomenon of the synchronisation of flagella in *C. reinhardtii* and the oscillatory flows induced by the swimming micro-organisms [39]. The Kuramoto model is a classical model used to describe synchronisation phenomena [40]. In the presence of external noise acting on the oscillators, such as noisy fluctuations in the velocity field in our case, the onset of synchronisation describing the appearance of a macroscopic mean field in a population of noisy coupled oscillators depends on the balance between the coupling strength and the noise intensity. Noise sets an upper cut-off on the distance between oscillators to sustain synchronisation [41]. This cut-off will change due to the dependence of noise on the phototactic guiding field strength, allowing synchronisation to occur also at a lower density in the presence of phototaxis. In addition to these mechanical interactions, biochemical signalling may also contribute to cell synchronisation at short distances. Chlorophyte algae conserved the calcium signalling mechanisms typical of eukaryotic cells [42]. Calcium is known to influence flagellar waveform and function in *C. reinhardtii* [43], especially during the phototactic orientation [44], which involves voltage-dependent calcium channels. The local variation of calcium may couple membrane depolarisation due to cellular proximity, a mechanism similar to that at work in calcium waves in tissues. For algal flagella it has been proposed that both mechanical and chemical interactions may be involved in the propagation of calcium waves. The presence of a mechanosensitive calcium channels at the base of the flagella of the alga [45] may support the theory of mechanically propagated calcium waves [46]. In this case, hydrodynamic coupling will exert mechanical forces on the neighbouring cell membrane causing calcium influx and the consequent flagellar activation.

In conclusion, the noise dominated phototactic reorientation mechanism driving the velocity field of the micro-swimmers to a polar ordered state resembles various other noisy driven collective motions and shares similarities with some phenomenological flocking models [32]. Thus, we think that the existence of a density threshold for the onset of polar order found here could be extended and used to explain the widespread ordered responses of clusters formed by living organisms.

## Materials and Methods

### Cell Culture and Setup

The wild-type biflagellate green alga *Chlamydomonas reinhardtii* was used for the experiments. The algae cell bodies are slightly prolate spheroids approximately 10 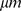 in diameter, swimming at an average speed of 50 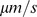 in free media. Synchronised algae cultures were grown in tris-acetate phosphate medium (TAP) using a 14/10 hours light/dark cycle. Cells were harvested for experiments at midlogarithm phase. A higher density was obtained gently centrifuging of cells suspensions for 4 minutes and leaving them at rest an hour for recovery. The experimental setup used for photo-movement assay consists of a poly-dimethylsiloxane (PDMS) microfluidic channel used to confine algae suspensions and an LED light source (Luxeon 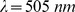) positioned at the end of the channel that is able to induce negative phototaxis (when turned on at 2V). PDMS microfluidic channels of 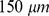 height and 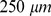 width were obtained through photolithographic methods and successively passivated with bovin serum albumin protein (5%) to prevent cell adhesion. Cell movement experiments were performed both in presence (light) and absence (dark) of the external phototactic light stimulus at different density suspensions and from different cultures (see Videos S1 and S2). Suspensions were imaged in bright-field microscopy using an actinic red light (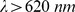) with x6 magnification objective (NA 0.1) (Fig. 1) and movies were recorded at 30 frame/sec.

### Data Analysis

Analysis was performed using a cross-correlation particle image velocimetry (PIV) technique [47] to extract the velocity field 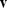 from consecutive movie frames. The interrogation window was 64 pixels wide and a spatial overlap of 75% was chosen to ensure sufficient image sampling. Each window represents nearly 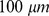 (an order of magnitude wider than the cell diameter) and defines a local spatial domain of the image over which correlation analysis was performed. The algorithm returns a matrix with velocity field elements represented in vectorial form 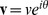, a velocity modulus 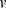 and an orientational polar angle 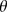 for each interrogation window. A moving average is applied to filter the PIV output from outliers. The corresponding cell density field was obtained by rescaling the amount of pixels occupied by cells by the pixel to cell area ratio.

The time autocorrelation function of the velocity direction shows the time evolution of the velocity correlation of a fixed spatial domain over time 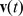 with respect a time reference 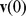 and is defined as
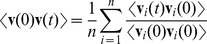
(5)where n is the number of the spatial domains within the image and 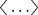 denotes an average over a set of starting times. The spatial correlation function is an auto-correlation function of the pair distance of the velocity belonging to the spatial domain 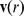 and the velocity 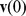 at the spatial domain reference,
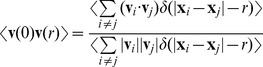
(6)where 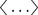 denotes an average over a set of a spatial domains. Using a similar notation, the spatial correlation of the fluctuation 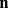 is defined as



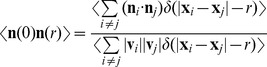
(7)To probe the local coherence of the directional motion of the orientation field the order parameter 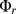 is used as presented elsewhere [22]. This term describes the average scalar product or alternatively the average cosine of the velocity vectors belonging to a region of radius r,
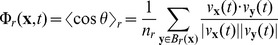
(8)where 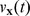 is the PIV extracted velocity field and 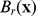 is a quasi-circular region of radius r, centered at 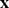 location, containing 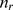 interrogation area elements. This term has a maximum value of unity only if the velocity vectors are parallel in the quasi-circular region 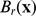.

## Supporting Information

Video S1
**Negative phototaxis.** Negative phototactic light 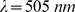 causes algae movement in a direction opposite to the light source stimulus (green arrows) (top). The algae were light adapted before experiments. Cell density distribution 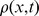 along the microfluidic channel main axis (bottom). Net-mass transport induced by phototaxis causes change of the algae accumulation from one end of the channel to the other.(AVI)

Video S2
**Absence of phototaxis.** Incoherent cells motion in absence of phototactic light stimulus (top). Cell density distribution 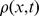 along the microfluidic channel main axis remains unaltered in space and time (bottom).(AVI)
